# Pharmacology and therapeutic potential of agarwood and agarwood tree leaves in periodontitis

**DOI:** 10.3389/fphar.2024.1468393

**Published:** 2024-09-11

**Authors:** Chen Xie, Jing-Zhe Dong, Bing-Shuai Lu, Peng-Yao Yan, Yun-Shan Zhao, Xin-Yue Ding, Cheng-En Lv, Xu Zheng

**Affiliations:** ^1^ Department of Stomatology, The First Affiliated Hospital of Hainan Medical University, Haikou, China; ^2^ School of Stomatology, Hainan Medical University, Haikou, China; ^3^ Integrated Department, Hainan Stomatological Hospital, Haikou, China

**Keywords:** agarwood, agarwood tree leaves, pharmacological action, mechanism, periodontitis, nanoemulsions

## Abstract

The main bioactive components of agarwood, derived from Aquilaria sinensis, include sesquiterpenes, 2-(2-phenethyl) chromone derivatives, aromatic compounds, and fatty acids, which typically exert anti-inflammatory, antioxidant, immune-modulating, hypoglycemic, and antitumor pharmacological effects in the form of essential oils. Agarwood tree leaves, rich in flavonoids, 2-(2-phenethyl) chromone compounds, and flavonoid compounds, also exhibit significant anti-inflammatory, antioxidant, and immune-modulating effects. These properties are particularly relevant to the treatment of periodontitis, given that inflammatory responses, oxidative stress, and immune dysregulation are key pathological mechanisms of the disease, highlighting the substantial potential of agarwood and agarwood tree leaves in this therapeutic area. However, the low solubility and poor bioavailability of essential oils present challenges that necessitate the development of improved active formulations. In this review, we will introduce the bioactive components, extraction methods, pharmacological actions, and clinical applications of agarwood and agarwood tree leaves, analyzing its prospects for the treatment of periodontitis.

## 1 Introduction

Periodontitis is an inflammatory disease of the periodontal tissues with plaque as the initiating factor and the host immune response as the promoting factor, which can cause clinical symptoms such as bleeding gums, periodontal pocket formation, alveolar bone resorption, loose and shifting teeth and even tooth loss. The 1999 Classification of Periodontal Diseases divides periodontal disease into gingivitis and periodontitis. The 2017 International Classification Symposium on Periodontal and Peri-implant Diseases addresses the revision of the 1999 Classification of Periodontal Diseases by simplifying the classification of periodontitis and adding staging and grading of periodontitis, which will help to make the diagnosis of periodontitis more precise and provide individualized diagnostic and therapeutic strategies for patients with periodontitis. In the new 2018 classification of periodontitis, the clinical definition is a microbial-associated, host-mediated inflammatory response to loss of periodontal attachment. The clinical diagnostic criteria are the presence of adjacent clinical attachment loss (CAL) on two or more teeth that are not adjacent to each other or the presence of buccal or lingual CAL ≥3 mm with periodontal pockets ≥3 mm on the buccal or lingual side of 2 or more teeth. The diagnosis of periodontitis can be confirmed by fulfilling one of these criteria (Tonetti and Sanz, 2019). Nowadays, periodontitis has been classified as a major dental disease by the World Health Organization (WHO) and is a public health problem that affects the oral and systemic health of the global population. Studies have shown that the global prevalence of chronic periodontitis is approximately 11.2%, making it the sixth most prevalent disease in the world, with approximately 743 million people worldwide suffering from chronic periodontitis (Marcenes et al., 2013). The prevalence of periodontitis increases with age, with the highest incidence in the 30–40-year-old age group, and with the aging of the global population, the prevalence of periodontitis is not encouraging (Kassebaum et al., 2014). The results of China’s fourth national oral epidemiology survey in 2017 showed that approximately 90% of adults suffer from varying degrees of periodontitis, and the prevalence of severe periodontitis in middle-aged and elderly patients increases significantly with higher age (Jiao et al., 2021). Due to the long-term inflammatory state of periodontal tissues, irreversible resorption of alveolar bone, resulting in tooth loosening and displacement, is the main cause of tooth loss in adults, resulting in a lower quality of life for patients and an increased financial burden of oral health maintenance.

Periodontitis can not only destroy periodontal tissues, but its dominant pathogenic bacteria, bacterial products and inflammatory mediators of periodontal tissues could reach the systemic organs with the help of blood circulation. A large body of evidence demonstrates that periodontitis is closely related to systemic diseases such as diabetes mellitus, cardiovascular disease, chronic obstructive pulmonary disease, chronic nephritis, and other systemic diseases (Chapple et al., 2013) and that the specific mechanisms may be related to metastatic infections, inflammatory responses, inflammatory mediators, and adaptive immune responses (Sanz et al., 2020). Periodontitis, the sixth most common complication of diabetes mellitus, microinflammation of periodontal tissues can affect glycemic control (Linden et al., 2013). Besides, a hyperglycemic environment, advanced glycosylation end-products, and hyperlipidemia can also exacerbate the inflammatory response of periodontal tissues (Sharma et al., 2016). There was a correlation between the severity of periodontitis and atherosclerotic plaque thickness, and the presence of Porphyromonas gingivalis was detected within the atherosclerotic plaque (Van Dyke and van Winkelhoff, 2013). Serum concentrations of C-reactive protein and low-density lipoprotein were elevated in patients with periodontitis, and expression levels of cardiovascular risk markers decreased after periodontal non-surgical treatment (Saini et al., 2011).

Nowadays, the treatment of periodontitis is mainly based on mechanical treatment, such as basic periodontal treatment and periodontal surgical treatment, supplemented by chemical medication, such as minocycline hydrochloride, metronidazole, chlorhexidine, doxycycline (Cai et al., 2024). However, the long-term use of antimicrobial drugs for periodontitis treatment is prone to adverse reactions such as drug resistance phenomenon and dysbiosis. In contrast, natural drug treatment has the advantage of fewer adverse reactions, so the search for natural drug treatment for periodontitis has turned out to be a new research direction.

In inflammatory treatment, phytotherapy can exert anti-inflammatory effects by modulating inflammatory mechanisms and releasing inflammatory mediators (Chang et al., 2013). Agarwood refers to the resin component formed after the Aquilaria or Gyrinops tree of the Thymelaeaceae family has been injured by the outside world, whose effective components are mainly composed of sesquiterpenes, 2-(2-phenylethyl) chromones, and aromatic components, with a wide range of pharmacological effects, such as anti-inflammatory, antioxidant, anti-bacterial, anti-tumor, and other effects (Muluke et al., 2016). Advantages of agarwood oil also exist in aromatherapy for mental disorders such as depression, anxiety, and insomnia (Zheng et al., 2018). In traditional Chinese medicine, agarwood is also used to alleviate symptoms such as chest and abdominal distension, gastric cold vomiting and dyspnea. In this article, we review the existing literature on the effective ingredients, extraction methods, pharmacological effects, and clinical applications of agarwood, summarizing the latest research in agarwood.

## 2 Major chemical composition of agarwood

Agarwood is commonly found in tropical forested areas, mainly in Southeast Asia, Southern China, and Indonesia. Globally, there are about 13 species of trees that can produce agarwood after external damage. These species include A. baillonii, A. beccariana, A. crassna, A. filaria, A. hirta, A. khasiana, A. malaccensis, A. microcarpa, *A. rostrata*, A. rugosa, A. sinensis, A. subintegra and *A. yunnanensis* (Lee and Mohamed, 2016). Agarwood owns a wide range of pharmacological effects, whose chemical composition are mainly made up of sesquiterpenes, 2-(2-phenylethyl) chromones, and aromatics (Pavlic et al., 2021).

### 2.1. Sesquiterpenoid

Sesquiterpenoids, as one of the active ingredients of agarwood, are formed by three isoprene units, and its content in agarwood extracts is high, accounting for 52% of the chemical composition of agarwood (Chen et al., 2012). It can be regarded as a criterion for grading the quality of agarwood. More than 210 sesquiterpenes have been extracted from agarwood, which can be categorized into eight main types (Smiley et al., 2015). Sesquiterpenes from agarwood exhibit a wide range of pharmacological effects, including anti-inflammatory, anti-tumor, antibacterial activities, inhibition of glycosidase, and regulation of the central nervous system (Wang Y. et al., 2022; Chen X. et al., 2022; Yang et al., 2019). A study by Xie demonstrated that sesquiterpenes from agarwood have significant anti-inflammatory activity, with an IC50 value for NO in lipopolysaccharide-induced RAW264.7 cells ranging from 5.46 to 14.07 μM (using aminoguanidine as a positive control with an IC50 of 20.33 ± 1.08 μM) (Xie et al., 2021). Huang’s research showed that sesquiterpenes from agarwood are effective against tumors and malaria (Huang X. L. et al., 2023). Additionally, the 2-(2-phenylethyl) chromone-sesterterpene hybrid compounds found in agarwood have protective effects on gastric mucosal cells and may be used to treat renal fibrosis by inhibiting the phosphorylation of Smad 3 (Huang et al., 2022; Zhang et al., 2023).

### 2.2 2-(2-Phenylethyl)chromone

2-(2-Phenylethyl) chromone is synthesized through the substitution of the phenylethyl moiety at the C-2 position of the chromogenic ketone, which accounts for 41% of the content in agarwood (Lee and Mohamed, 2016). The high cost of obtaining agarwood samples and chromone standards presents a significant challenge in the identification of agarwood chromone derivatives. To address this, Du has developed an enhanced data integration and filtering strategy that provides a detailed characterization and summary of the characteristic fragments and cleavage rules for 2-(2-phenylethyl) chromone monomers and their dimers, which introduces a novel research approach for the identification of agarwood chromone monomers and dimers (Li W. et al., 2021). As a principal bioactive constituent of agarwood, 2-(2-Phenylethyl)chromone exhibits a wide range of therapeutic properties, including anti-inflammatory, anti-tumor, neuroprotective functions, and the inhibition of acetylcholinesterase and glucosidases (Chen et al., 2012). The 2-(2-phenylethyl)chromone derivatives extracted from agarwood exhibit significant inhibitory effects on α-glucosidase, with IC50 values ranging from 7.8 ± 0.3 to 137.7 ± 3.0 μM (Acarbose, 743.4 ± 3.3 μM; Genistein, 8.3 ± 0.1 μM) (Wang Y. et al., 2022). Sarmah utilized computational simulation methods such as molecular docking, absorption, distribution, metabolism, excretion, toxicity, and molecular dynamics to discover that 2-(2-phenylethyl) chromone from agarwood can efficiently inhibit cyclooxygenase (COX) and lipoxygenase (LOX), with a higher affinity compared to ibuprofen, which suggests its potential as an effective inhibitor for the treatment of inflammatory diseases (Yang et al., 2019). Agarwood possesses unique advantages in the treatment of cardiovascular diseases. Derivatives of 2-(2-phenylethyl) chromone have been shown to mitigate the expression of CD36 in macrophages mediated by endoplasmic reticulum stress, thereby inhibiting the formation of foam cells and exerting a therapeutic effect on atherosclerosis (Chen L. et al., 2022). This mechanism of action represents a promising approach in the management of cardiovascular conditions, where the regulation of cellular processes and inflammation plays a pivotal role. The ability of these chromone derivatives to modulate key pathways involved in atherosclerotic plaque development highlights their potential as novel therapeutic agents. In addition, research by Ma has demonstrated that agarwood extracts rich in 2-(2-phenylethyl)chromone can ameliorate taurocholic acid-induced apoptosis in gastric epithelial cells by modulating endoplasmic reticulum stress, thereby exhibiting therapeutic efficacy against bile reflux gastritis (Xie et al., 2021). This finding underscores the potential of 2-(2-phenylethyl) chromone-enriched agarwood extracts in treating gastrointestinal conditions, particularly those characterized by cellular damage and inflammatory responses.

### 2.3 Low-weight aromatic compounds (LACs)

Agarwood has a long-standing history in aromatherapy (Huang X. L. et al., 2023), where its unique fragrance emitted upon heating is known for its beneficial effects on sleep disorders. The smoke from burning agarwood contains a significant amount of LACs, which, when inhaled, can aid in sleep induction and improvement (Huang et al., 2022). Benzyl acetone is a key component responsible for the sedative effects (Zhang et al., 2023). Research by Castro has indicated that while LACs from agarwood exhibit sedative potential when inhaled individually, the combined use of these compounds results in a diminished sedative effect (Castro and Ito, 2021). This reduction in efficacy may be attributed to antagonistic actions due to the structural similarities among the LACs (Table 1).

**TABLE 1:** Summary of species and chemical composition of Agarwood.

Chemical composition	Species	Source	Extraction method	Pharmacology	References
Sesquiterpenoid	Aquilaria walla	Thailand	Extracted with ethyl ether (1.5 L)	Inhibition of α-glucosidase (IC50 = 112.3 ± 4.5 μM) (acarbosec, IC50 = 743.4 ± 3.3 μM)	Yang et al. (2019)
	Aquilaria sinensis	China	95% EtOH (150 L, flow rate: 4.5 L/h)	Anti-caner effects MCF-7 cells: IC50 = 2.834 ± 1.121 μM MDA-MB-231 cells: IC50 = 1.545 ± 1.116 μM normal liver cells LO2: IC50 = 27.82 ± 1.093 μM	Chen et al. (2022a)
	Aquilaria agallocha	Vietnam	95% ethanol (3 × 20 L, 2 h each time)	Anti-inflammatory effects NO production IC50 = 5.46–14.07 μM (Aminoguanidine, IC50 = 20.33 ± 1.08 μM)	Xie et al. (2021)
	Aquilaria sinensis	China	95% EtOH (150 L, flow rate: 4.5 L/h)	Downregulating proliferation and migration activity of KYSE30 and BGC-823 cells Plasmodium falciparum 3D7 strain, IC50 = 21.67 ± 1.25 μM (Artemisinin, IC50 = 559.4 ± 66.55 μM)	Huang et al. (2023b)
	Aquilaria sinensis	China	95% EtOH (3 × 120 L, each 2.0 h)	Protecting against gastric mucosal cell insult	Zhang et al. (2023)
	Aquilaria sinensis	China	95% EtOH (150 L, flow rate: 4.5 L/h)	inhibiting the phosphorylation of Smad 3	Huang et al. (2022)
2-(2-Phenylethyl)chromone	Aquilaria sinensis	China	95% ethanol (70 L)	Anti-inflammatory effects NO production IC50 = 5.60 ± 0.93 μM (Indomethacin, IC50 = 31.23 ± 1.28 μM)	Hu et al. (2024)
	Aquilaria sinensis	China	95% EtOH (50.0 L × 3)	Anti-caner effects SMMC-7721, IC50 = 18.82 μg/mL MGC-803, IC50 = 25.35 μg/mL OV-90, IC50 = 31.60 μg/mL	Liu et al. (2020b)
	Aquilaria walla	Thailand	ethyl ether (1.5 L) 95% EtOH (2.5 L × 5) ethyl acetate (2.0 L × 3)	Anti-caner effects K562: IC50 = 13.40–28.96 μM BEL-7402: IC50 = 15.49 ± 0.09 μM SGC-7901: IC50 = 22.08 ± 0.06 μM A549: IC50 = 28.96 ± 0.40 μM Hela: IC50 = 22.19 ± 0.33 μM	Chen et al. (2023)
	Aquilaria filaria	Indonesia	95% ethanol (5.0 L × 6)	Inhibition of α-glucosidase IC50 = 7.8–137.7 μM (Acarbose, IC50 = 743.4 ± 3.3 μM; Genistein, IC50 = 8.3 ± 0.1 μM)	Yang et al. (2019)
	Aquilaria sinensis	China	95% EtOH (2 × 100 L, each 2.0 h) and 70% EtOH (100 L, 2.0 h)	Downregulating therosclerotic lesions in the ApoE−/−mice	Ma et al. (2024)
	Aquilaria sinensis	China	95% EtOH (2 × 100 L, each 2.0 h) and 70% EtOH (100 L, 2.0 h)	Protecting against taurocholic acid-induced gastric epithelial cells apoptosis	Ma et al. (2022)
Low-Weight Aromatic Compound	Not specified	Japan	Hydro-distillation	Enhancing the fragrance of agarwood	Takamatsu and Ito (2018)
	Not specified	China	Steam distillation	Sedative and hypnotic effects	Wang et al. (2022a)
	Not specified	Japan	Heated	Sedative effects	Castro and Ito (2021)

## 3 Induction methods of agarwood

Due to its unique aromatic odor and valuable medicinal value, agarwood is widely demanded in the world, which leads to its high price (Naziz et al., 2019). Agarwood is composed of resin secreted by trees after the outside world has stimulated them, leading to the scarcity of wild agarwood, coupled with its high price, exacerbating the uncontrolled logging of wild agarwood, making the survival of agarwood trees even more serious (Azren et al., 2018). Thus, it is particularly important to find ways to produce agarwood sustainably.

The formation pathways of agarwood are primarily divided into natural occurrence and artificial induction. In the natural process, agarwood is often formed when a tree is struck by lightning, bitten by animals, or invaded by microorganisms, leading to the formation of wounds that trigger the tree’s defense response and subsequent resin secretion. The advantage of this method is that it can produce high-quality agarwood without the need for human intervention and at a low cost. However, the disadvantages are also apparent: the long time required for agarwood formation, low yield, inconsistent quality, and the increased risk of indiscriminate harvesting of wild trees (Azren et al., 2018).

### 3.1 Traditional induction methods

As mentioned above, the natural formation of agarwood has several drawbacks, making the technology of artificial induction of agarwood a key solution to address the issues mentioned above, with the aim of increasing agarwood production to meet market demand. With the advancement of technology, the quality of artificially induced agarwood has significantly improved. Research by Ma has shown that the chemical composition of artificially induced agarwood is similar to that of wild agarwood, and its antioxidant properties, as well as its ability to inhibit glucosidase and acetylcholinesterase, are comparable to those of wild agarwood (Ma et al., 2023).

The artificial induction of agarwood formation is primarily categorized into traditional methods, biological induction, and chemical induction (Yan et al., 2019). Traditional methods involve creating wounds on the tree through manual drilling, hole-making, burning, or chopping to simulate the natural conditions under which a tree would be injured, thereby inducing resin secretion and the formation of agarwood. The advantage of these methods is that they do not require guidance from specialized technical personnel. However, the downside is that agarwood is formed only locally at the site of injury, leading to inconsistent yields and average quality (Azren et al., 2018). Xu’s research on the dynamic response of agarwood to mechanical damage has revealed the molecular mechanisms behind agarwood formation, providing a new reference for the quality assessment of agarwood (Xu et al., 2023).

### 3.2 Biological induction methods

Artificial biological induction involves drilling into the tree and implanting biological inducers to simulate the natural infection of trees. However, not all microorganisms are capable of inducing healthy agarwood trees to form agarwood. Commonly used fungal species include Fusarium, Lasiodiplodia, Penicillium, and Aspergillus (Chen et al., 2017; Sangareswari Nagajothi et al., 2016). Phaeoacremonium rubrigenum can promote the accumulation of sesquiterpene compounds in agarwood by inducing plant host phosphorylation (Liu et al., 2022). Huang has demonstrated that agarwood induced artificially using fungal inducers prepared from wild agarwood is similar in quality to wild agarwood (Huang M. et al., 2023). Wu has developed the Biologically Agarwood-Inducing Technique (Agar-Bit), where biological inducers are injected into the heartwood part of the agarwood tree (Wu and Yu, 2023). This method can induce agarwood formation within 6 months while maintaining the health and vitality of the tree, offering a new approach to whole-tree agarwood induction. The advantages of biological induction include low cost and ease of operation. However, the downside is the potential inconsistency in agarwood quality due to variations in microbial populations at the inoculation sites (Naziz et al., 2019).

### 3.3 Chemical induction methods

When it was discovered that certain signaling molecules could trigger the defense response of agarwood trees, promoting the secretion of resin components in healthy agarwood to resist external stimuli, chemical induction became a new research hotspot (Wu et al., 2024). Chemical induction can solve the problem of poor repeatability in the quality of agarwood formed through biological induction, and it appears to be the most ideal and reliable alternative to natural agarwood formation.

Chemical induction can solve the problem of poor reproducibility of agarwood formation quality in biological induction and seems to be the most ideal and reliable alternative to natural agarwood formation. Chemical induction refers to the direct application of chemical agents such as ferrous chloride, hydrogen peroxide, and methyl jasmonate to trees to induce agarwood formation. For example, hydrogen peroxide promotes programmed cell death in agarwood and the accumulation of salicylic acid and sesquiterpene compounds (Lv et al., 2019; Liu et al., 2015). Methyl jasmonate promotes the formation of sesquiterpene compounds in agarwood (Xu et al., 2016; Sun P. W. et al., 2020). Currently, several techniques for chemical induction of agarwood have been developed, such as the Cultivated Agarwood Kit (CA-kit), Biological Agarwood Induction Technology (Agar-bit), and Whole-Tree Agarwood Induction Technology (Agar-Wit) (Tan et al., 2019). The advantages of chemical induction include convenient operation, stable and reliable agarwood quality, and the ability to induce the entire tree. However, the downside is that it can easily cause tree rot, high concentrations of chemical agents can cause the death of the tree, and the potential environmental hazards of chemical agents must still be considered (Azren et al., 2018; Faizal et al., 2021). Therefore, before the large-scale promotion of the chemical induction method, extensive experiments are still needed to verify its safety and feasibility repeatedly (Table 2).

**TABLE 2:** Summary of induction methods of Agarwood.

Method	Concept	Merit	Demerit	References
Natural	When a tree is struck by lightning, bitten by animals, or invaded by microorganisms, leading to the formation of wounds that trigger the tree’s defense response and subsequent resin secretion	Produce high-quality agarwood Low cost without the need for human intervention	Low yield Inconsistent quality Increased risk of indiscriminate harvesting of wild trees Waste time	Azren et al. (2018)
Artificial traditional	Creating wounds on the tree through manual drilling, hole-making, burning, or chopping to simulate the natural conditions under which a tree would be injured, thereby inducing resin secretion and the formation of agarwood	Without specialized technical personnel	Inconsistent yields average quality	Azren et al. (2018), Xu et al. (2023), Yan et al. (2019)
Artificial biological	Drilling into the tree and implanting biological inducers to simulate the natural infection of trees	Low cost Ease of operation	Inconsistent quality of agarwood	Chen et al. (2017), Sangareswari Nagajothi et al. (2016), Wu and Yu (2023), Naziz et al. (2019)
Artificial chemical	The direct application of chemical agents such as ferrous chloride, hydrogen peroxide, and methyl jasmonate to trees to induce agarwood formation	Convenient operation Stable and reliable agarwood quality The ability to induce the entire tree	Easily cause tree rot High concentrations of chemical agents can cause the death of the tree	Azren et al. (2018), Lv et al. (2019), Sun et al. (2020a), Xu et al. (2016)

## 4 Methods of agarwood essential oil extraction

The pharmacological effects of agarwood are extensive, and its active ingredients often exist in the form of essential oil. However, since the extraction process of agarwood essential oil is not the primary focus of this article, a brief introduction to the relevant extraction methods will be provided below. Traditional methods of agarwood essential oil extraction include steam distillation and hydro-distillation, both of which are based on the principle that water and essential oil are incompatible with each other to achieve the purpose of separation and purification of essential oil. However, they have been gradually replaced by new extraction methods due to the shortcomings of long time-consuming, low production capacity and high energy consumption (Alamil et al., 2022). In contrast, supercritical fluid extraction and subcritical water extraction are recognized for their high efficiency, stable quality, and the richness of active components they yield (Ibrahim et al., 2011). Research by Tian has demonstrated that agarwood oil extracted using supercritical extraction exhibits superior antioxidant activity and antimicrobial effects compared to that obtained through steam distillation (Tian et al., 2019). At a concentration of 10 g/L, the agarwood essential oil extracted by supercritical fluid extraction achieved a scavenging rate of 96.2%, while steam distillation only reached a scavenging rate of 67.7%. Regarding the DPPH radical scavenging ability, the essential oil of agarwood extracted by supercritical fluid extraction showed an increase in the scavenging rate from 33.69% to 92.15% at concentrations ranging from 1 to 10 g/L. In contrast, the essential oil extracted by steam distillation reached a maximum scavenging rate of only 40.98% at concentrations between 8 and 14 g/L. Samadi conducted a comparison between hydro-distillation and subcritical water extraction methods for agarwood essential oil extraction and found that subcritical water extraction can produce agarwood essential oil of higher quality with a greater abundance of active components (Samadi et al., 2020). Analysis with GC-MS revealed that HD, involving a 1-week soaking period followed by 16 h of distillation, yielded an essential oil from which 43 chemical constituents could be identified. In contrast, the essential oil extracted using the SCWE method, which took only 13 min, contained 50 identifiable chemical constituents. The proportion of bioactive components such as sesquiterpenes and chromone compounds was higher in the essential oil obtained by SCWE than in HD. Additionally, the essential oil extracted via the SCWE method contained a greater variety of bioactive compounds, including guaiacol, syringol, furfural, vanillin, and phenylacetaldehyde.

## 5 Pharmacological effects of agarwood

**FIGURE 1:**
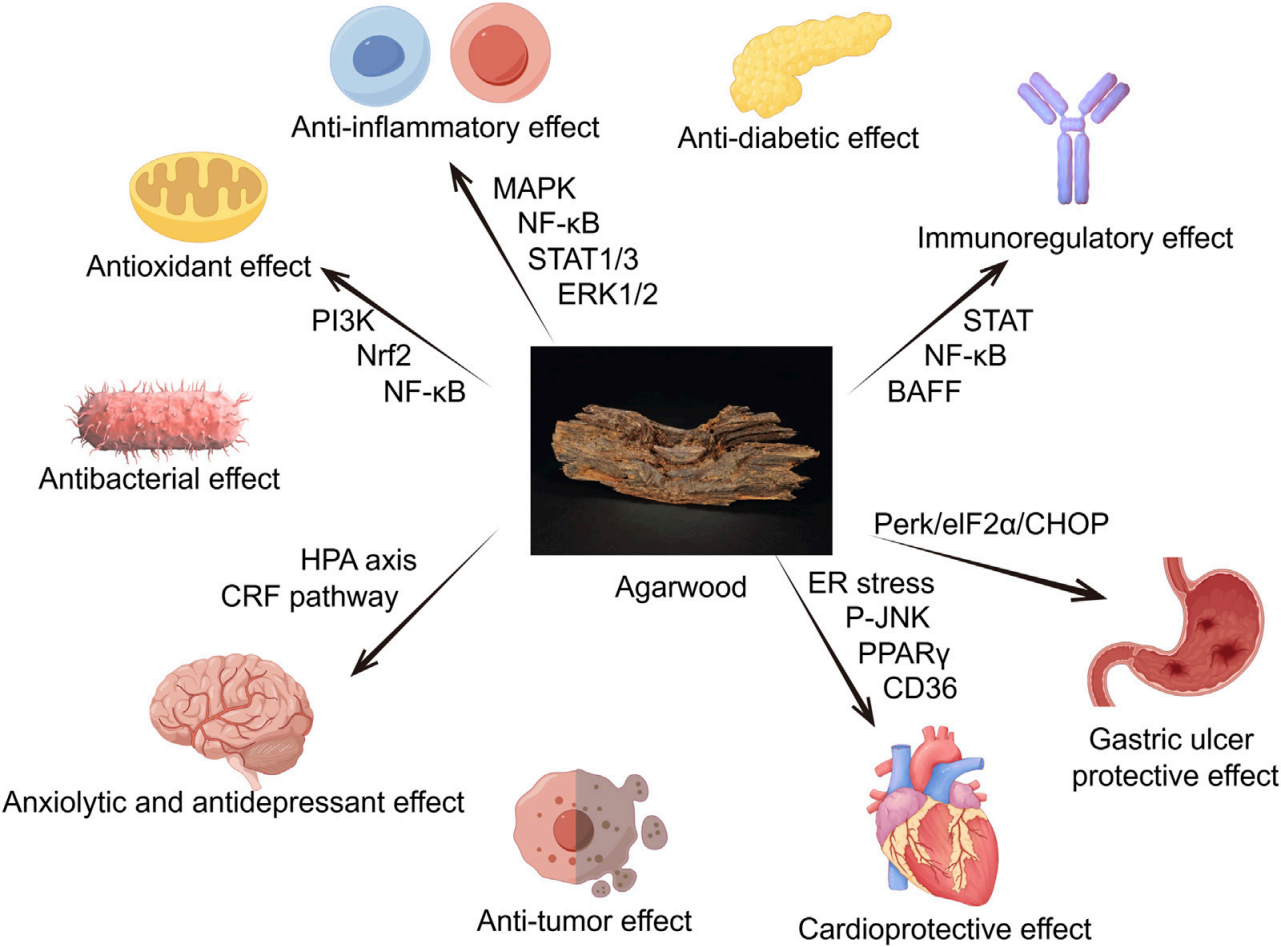
Agarwood has a positive effect on multiple organs and physiological activities throughout the body, such as anti-inflammatory, antioxidant, antibacterial, anxiolytic and antidepressant, anti-tumor, anti-diabetic, acetylcholinesterase inhibition and other effects (By Figdraw).

Due to its richness in sesquiterpenes, 2-(2-phenylethyl) chromones, aromatic compounds and its potent anti-inflammatory, antioxidant, antibacterial, antitumor, sedative and analgesic pharmacological effects, agarwood is commonly used to treat cardiovascular, nervous system, digestive, and respiratory diseases. The following sections will introduce its pharmacological effects (Figures 1).

### 5.1 Anti-inflammatory effect

Inflammatory response refers to maintaining homeostasis by eliminating pathogenic microorganisms through intrinsic and adaptive immune responses when pathogenic microorganisms stimulate the organism. There has been a significant amount of research on the anti-inflammatory effects of agarwood essential oil against inflammatory diseases, and the anti-inflammatory effects of agarwood are attributed to its ability to modulate the production of inflammatory mediators and reduce oxidative stress, which are critical factors in the pathogenesis of various inflammatory diseases (Alamil et al., 2022). As previously mentioned, sesquiterpenes and 2-(2-phenylethyl) chromone compounds exhibit potent anti-inflammatory activities. Numerous vitro studies have demonstrated that sesquiterpene compounds isolated from agarwood can effectively inhibit the release of nitric oxide by macrophages induced by lipopolysaccharide (Xie et al., 2021; Zhao et al., 2016; Hu et al., 2024). However, the specific mechanisms by which sesquiterpene compounds exert their anti-inflammatory effects remain to be elucidated. In contrast, 2-(2-phenylethyl) chromone compounds have been shown to suppress the release of nitric oxide in RAW 264.7 cells during inflammation by inhibiting the activation of NF-κB without cytotoxicity (Wang S. L. et al., 2018; Yu et al., 2020; Yang et al., 2023). Additionally, 2-(2-phenylethyl)chromone compounds can inhibit the release of inflammatory mediators such as NO, TNF-α, IL-6, IL-1β, and PGE2 by suppressing the STAT and MAPK signaling pathways (Zhu et al., 2016a). Gao administered agarwood essential oil via gavage, effectively suppressing ear edema induced by dimethyl benzene and foot edema caused by carrageenan (Gao et al., 2019). This anti-inflammatory effect may be associated with the inhibition of p-STAT3 protein expression. In addition, agarwood extracts have been shown to provide protective effects against gastric ulcers and intestinal mucositis by suppressing inflammatory responses and alleviating anxiety symptoms (Wang C. et al., 2021; Zheng et al., 2019; Wang S. et al., 2018). Inflammatory responses are closely related to conditions such as cancer and immune reactions, and agarwood has demonstrated inhibitory effects on various inflammatory diseases, which could potentially validate the broad pharmacological activities of agarwood from a different perspective (Table 3).

**TABLE 3:** Anti-inflammatory effects of Agarwood.

Compound	Experiment	Pathways	Results	References
Sesquiterpenes	In vitro- LPS-induced RAW 264.7	Not specified	↓ NO production	Xie et al. (2021)
2-(2-phenylethyl)chromones, sesquiterpenes	In vitro- LPS-induced RAW 264.7	Not specified	↓ NO production	Hu et al. (2024)
Sesquiterpenoid	In vitro- LPS-induced RAW 264.7	Not specified	↓ NO production	Zhao et al. (2016)
2-(2-Phenylethyl)-4H-chromen-4-one	In vitro- LPS-induced RAW 264.7	↓ NF-κB activation	↓ NO production	Wang et al. (2018b)
5, 6, 7, 8-tetrahydro-2-(2-phenylethyl)chromones	In vitro- LPS-induced RAW 264.7	Not specified	↓ NO production	Yu et al. (2020)
2-(2-phenylethyl)chromone	In vitro- LPS-induced RAW 264.7	Not specified	↓ iNOS, COX-2	Yang et al. (2023)
2-(2-phenethyl)-chromone	In vitro- LPS-induced RAW 264.7	↓ Phosphorylation of and ERK1/2 STAT1/3	↓ NO, iNOS, TNF-α, IL-1β, IL-6, PGE2, COX-2	Zhu et al. (2016a)
Agarwood Essential Oil	In vitro- LPS-induced RAW 264.7 In vivo-ICR Mice, Sprague Dawley mice	↓ STAT3 signaling pathway	↓ Ear swelling caused by xylene ↓ Foot swelling caused by carrageenan ↓ IL-1β, IL-6	Gao et al. (2019)
Agarwood Alcohol Extract	In vivo-ICR Mice	↓ Phosphorylation of NF-κB, MAPK	↓ The occurrence of gastric ulcers ↓ NO, IL-6, IL-10, IL-1β, ↑ GSH, SOD	Wang et al. (2021a)
Aquilariae Lignum Resinatum extract	In vivo-male Wistar rats	Not specified	↑ Intestinal mucosa recovery from damage caused by 5-Fuorouracil ↑ MDA, SOD ↓ COX-2, TNF-α	Zheng et al. (2019)

### 5.2 Antioxidant effect

Chronic respiratory diseases such as chronic obstructive pulmonary disease and asthma often exhibit pathological characteristics of oxidative stress and inflammatory responses. Agarwood essential oil, rich in functionally active components such as sesquiterpenes and 2-(2-phenylethyl) chromones, can be a potential source of antioxidant active materials. Chen’s research indicates that agarwood essential oil’s DPPH and ABTS radical scavenging abilities are positively correlated with concentration (Chen L. et al., 2022). At a 5 mg/mL concentration, agarwood essential oil obtained by supercritical extraction exhibited a DPPH radical scavenging rate of over 50%. At a concentration of 2 mg/mL, the ABTS radical scavenging rate ranged from 53.03% to 83.26%. Ma’s research shows that the DPPH and ABTS radical scavenging capabilities of Induced agarwood (IA) and wild agarwood (WA) are close (Ma et al., 2023). At a concentration of 0.8 mg/mL, the DPPH radical scavenging rates for IA and WA were 91.26% and 91.59%, respectively. At 0.2 mg/mL, the ABTS radical scavenging rates for IA and WA were 91.03% and 94.80%, respectively.

Due to the poor solubility and low bioavailability of agarwood essential oil, these factors have become barriers to its clinical application and promotion. To address this issue, Rubis utilized ultrasonication to prepare agarwood essential oil into a nanoemulsion, investigating its potential antioxidant and anti-inflammatory effects on airway basal epithelial cells under cigarette smoke extract stimulation. The experimental results showed that the agarwood nanoemulsion could inhibit oxidative stress by inducing the expression of antioxidant genes and exert protective effects by suppressing inflammatory responses, demonstrating the therapeutic potential of agarwood for chronic obstructive pulmonary disease (De Rubis et al., 2023). In further research, Malik prepared agarwood oil nanoemulsions and explored its antioxidant and anti-inflammatory capabilities on lipopolysaccharide-induced RAW264.7 macrophages. The results demonstrated that the agarwood oil nanoemulsions significantly reduced the production of reactive oxygen species (ROS) and the expression of the antioxidant gene heme oxygenase-1 (HO-1), revealing a strong antioxidant potential (Malik et al., 2023). In addition to the studies above, Wang prepared an animal model of alcoholic fatty liver disease and found that agarwood extract could effectively reduce the expression levels of alanine aminotransferase (ALT), aspartate aminotransferase (AST), triglyceride (TG), and cholesterol (CHO), lower blood lipid levels, and improve liver function through its antioxidant effects, alleviating the damage to the liver caused by a high-fat diet and alcohol consumption (Wang et al., 2023). Through *in vivo* studies on the effects of agarwood extracts on myocardial ischemia, Wang discovered that agarwood extracts could upregulate and activate the Nrf2-ARE pathway, exhibiting significant antioxidant effects, thereby playing a protective role against myocardial infarction (Wang et al., 2020) (Table 4).

**TABLE 4:** Antioxidant effects of Agarwood.

Compound	Experiment	Pathways	Results	References
Agarwood oil nanoemulsion	In vitro-cigarette smoke extract-treated BCi-NS1.1 airway basal epithelial cells	↑ PI3K pro-survival signalling pathway	↓ IL-10, IL-18BP, IFN-γ, TFF3, relaxin-2, VDBP, GH, PDGF ↓ GCLC, GSTP1	De Rubis et al. (2023)
Agarwood oil nanoemulsion	In vitro- LPS-induced RAW 264.7	Not specified	↓ ROS, NO, iNOS, TNF-α, IL-1β, IL-6, HO-1	Malik et al. (2023)
Agarwood extract	In vivo-male C57 mice	↓ Nrf2, NF-κB signalling pathway	↓ AST, ALT, TG, CHO ↓ NO, LPO, H2O2 ↑ CAT, SOD, T-AOC	Wang et al. (2023)
Agarwood Alcohol Extract	In vivo-male Sprague-Dawley	↑ Nrf2-ARE pathway ↓ Bcl-2 pathway	↓ CK, LDH, ALT, AST ↓ LPO, H2O2 ↑ T-AOC, CAT ↑ Nrf2, GST, HO-1 ↓ Bax, Bik, Bad	Wang et al. (2020)

### 5.3 Antibacterial effect

The antibacterial activity of agarwood is beneficial in combating bacterial and fungal infections. An increasing body of research *in vitro* has demonstrated that agarwood essential oil exhibits significant inhibitory effects against *Staphylococcus aureus* and *Bacillus subtilis* (Chen et al., 2011). Dahham isolated β-caryophyllene from agarwood essential oil and investigated its antimicrobial and antioxidant properties. *In vitro*, experimental results indicated that β-caryophyllene present in agarwood has notable antibacterial effects against *Bacillus subtilis*, *Staphylococcus aureus*, *Escherichia coli*, *Klebsiella pneumoniae*, and *Pseudomonas aeruginosa*. Moreover, its antifungal activity against Trichoderma reesei is superior to that of kanamycin (Dahham et al., 2015). Hu compared the antimicrobial activity of smoke produced by burning both Qi-Nan agarwood and ordinary agarwood (induced by the knife-cutting method), revealing that both demonstrated potent antimicrobial activity against *Escherichia coli*, *Pseudomonas aeruginosa*, and *Staphylococcus aureus*, with Qi-Nan agarwood showing greater efficacy than ordinary agarwood (Hu et al., 2023). Nurminah’s research suggested that agarwood distillate water extracts also possess inhibitory effects on *Streptococcus* mutans *in vitro* (Nurminah et al., 2021) (Table 5).

**TABLE 5:** Antibacterial effects of Agarwood.

Compound	Methods	Tested strains	Results	References
Agarwood Essential Oil	In vitro-agar well diffusion method	*E. coli*	MIC = 6.25 mg/mL	Chen et al. (2011)
		*S. aureus*	MIC = 0.195 mg/mL	
		*B. subtilis*	MIC = 0.195 mg/mL	
Sesquiterpene β-Caryophyllene	In vitro-disk diffusion method	B. cereus	MIC = 9 ± 1.1 μM	Dahham et al. (2015)
		*B. subtilis*	MIC = 8 ± 2.1 μM	
		*S. aureus*	MIC = 3 ± 0.4 μM	
		F. coli	MIC = 9 ± 2.2 μM	
		*K. pneumoniae*	MIC = 14 ± 2.7 μM	
		*P. aeruginosa*	MIC = 7 ± 1.2 μM	
		*A. niger*	MIC = 6 ± 0.8 μM	
		P. citrinum	MIC = 7 ± 1.2 μM	
		R. oryzae	MIC = 6 ± 0.5 μM	
		T. reesei	MIC = 4 ± 0.7 μM	
Cigarette gas of agarwood	In vitro-identification test of air disinfection effect	*S. aureus*	Killing rate = 99%	Hu et al. (2023)

### 5.4 Anxiolytic and antidepressant effect

Aromatherapy has long been utilized to relax the mind and body and relieve stress. Agarwood essential oil, known for its unique aromatic scent and practical components, is commonly used in the treatment of insomnia, anxiety, and depression, offering distinct advantages in the treatment of psychiatric disorders. Agarwood emits a distinctive fragrance when heated, and inhaling the smoke generated can help with sleep induction and improve sleep disorders (Wang C. et al., 2022). Agarwood essential oil is also calming by regulating the expression of GABA_A_ receptors and enhancing receptor function (Wang et al., 2017). Through experiments such as the light-dark box test and the tail suspension test, Wang found that agarwood essential oil may exert anxiolytic and antidepressant effects by inhibiting the release of corticotropin-releasing factor and the activation of the hypothalamic-pituitary-adrenal (HPA) axis, with effects similar to those of diazepam (2.5 mg/kg) (Wang S. et al., 2018). Li, through preparing a mixed essential volatile oil of Aquilaria sinensis (Lour.) Gilg and Aucklandia costus Falc, arrived at conclusions consistent with those of Wang’s research (Li H. et al., 2021). Using network pharmacology combined with solid-phase microextraction/gas chromatography-time of flight mass spectrometry, Pang discovered that sesquiterpenes in agarwood can exert anxiolytic effects by acting on multiple targets and signaling pathways (Pang et al., 2024). Han, through the injection of scopolamine to induce a model of learning and memory impairments, demonstrated significant alleviation of symptoms after inhalation of agarwood smoke (Han et al., 2021) (Table 6).

**TABLE 6:** Anxiolytic and Antidepressant effects of Agarwood.

Compound	Experiment	Pathways	Results	References
Agarwood Essential Oil	In vivo-male KM mice	↑ GABA, 5-HT nervous systems ↓ Glu nervous systems	↓ Sleep latency of mice ↑ Sleep time of the mice ↓ Autonomous activities ↑ GABAA, 5-HT, AD, ↓ Glu, GABAA/Glu ↑ 5-HT1A, GluR1, VGluT1	Wang et al. (2022a)
Agarwood Essential Oil	In vivo-ICR Mice	Not specified	↓ Total distance traveled ↑ The rate of sleeping induced by the subthreshold pentobarbital sodium ↓ The latency of sleeping time ↑ GABAα, GABAγ	Wang et al. (2017)
Agarwood Essential Oil	In vivo-male ICR Mice	↓ CRF pathway and the hyperactive HPA axis	↑ The time and distance in light compartment of mice of the Light Dark Exploration Test ↑ The time and distance spent in the central area of the Elevated Plus Maze Test ↓ The immobility of the Tail Suspension Test ↓ IL-6, IL-1α, IL-1β, ACTH, CORT ↓ The gene expression of nNOS, CRF, CRFR	Wang et al. (2018a)
Aquilaria sinensis (Lour.) Gilg and Aucklandia costus Falc. Volatile Oils	In vivo-Sprague Dawley mice	↓ HPA Axis Hyperactivity	↓ The ommobility time in Forced Swimming Test ↓ ACTH, CORT, CRH ↑ 5-HT, Ach, DA, NE, 5-HT_1A_	Li et al. (2021a)
Agarwood Incense	In vivo-male C57 mice	Not specified	↓ Scopolamine-induced memory and learning impairment in mice	Han et al. (2021)

### 5.5 Anti-tumor effect

Agarwood’s anti-tumor effects are of interest in oncology, as it may inhibit tumor growth and induce apoptosis in cancer cells. Studies have indicated that oral administration of agarwood essential oil at doses of 100 mg/kg and 500 mg/kg for 28 consecutive days had no significant impact on the vital signs of mice. However, it exhibited inhibitory effects on the growth of colon cancer cells (Dahham et al., 2016). As its active constituents, the sesquiterpenes and chromone compounds in agarwood have also been extensively studied for their cytotoxicity. A new sesquiterpene compound was isolated from Aquilaria sinensis, which exhibited strong sensitivity to breast cancer cells. The relevant mechanism may be promoting the expression of reactive oxygen species (ROS) in cells, which promotes cancer cell apoptosis. This compound could potentially become a promising anticancer agent (Chen L. et al., 2022). Utilizing LC-MS-guided fractionation, Chen obtained five new derivatives of 2-(2-phenylethyl) chromone, which significantly inhibited the growth of five types of human cancer cells (IC50 13.40–28.96 μM), with cisplatin serving as a positive control (IC50 3.08–11.29 μM) (Chen et al., 2023). Similar findings were observed in cytotoxicity studies of 2-(2-phenylethyl) chromone derivatives isolated from agarwood (Xia et al., 2019). β-Caryophyllene isolated from the essential oil of Aquilaria crassna effectively inhibited the proliferation, migration, invasion, and spheroid formation of colorectal cancer cells (Dahham et al., 2015) (Table 7).

**TABLE 7:** Anti-tumor effects of Agarwood.

Compound	Experiment	Pathways	Results	References
Agarwood Essential Oil	In vivo-Athymic NCR nu/nu nude mice	Not specified	↓ The subcutaneous tumor of HCT 116 colorectal carcinoma cells	Dahham et al. (2016)
Sesquiterpene and Triterpenoids from Agarwood	In vitro- MCF-7 cells, MDA-MB-231 cells, normal liver cells LO2	Not specified	MCF-7 cells: IC50 = 2.834 ± 1.121 μM MDA-MB-231 cells: IC50 = 1.545 ± 1.116 μM normal liver cells LO2: IC50 = 27.82 ± 1.093 μM	Chen et al. (2022a)
2-(2-phenethyl)chromone	In vitro- tumor cell lines (K562, A549, SGC-7901, BEL-7402, Hela)	Not specified	K562: IC_50_ = 13.40–28.96 μM BEL-7402: IC_50_ = 15.49 ± 0.09 μM SGC-7901: IC_50_ = 22.08 ± 0.06 μM A549: IC_50_ = 28.96 ± 0.40 μM Hela: IC_50_ = 22.19 ± 0.33 μM	Chen et al. (2023)
Dimeric 2-(2-phenylethyl)chromones and sesquiterpene-2-(2-phenylethyl)chromone conjugates	In vitro-tumor cell lines (K562, K549, SGC-7901 and Hela)	Not specified	IC50 = 10.93–49.0 μM	Xia et al. (2019)
Dimeric sesquiterpenoid-4H-chromone	In vitro-tumor cell lines (K562, SGC-7901, BEL-7402, A549, Hela)	Not specified	IC50 = 17.6–46.3 μM	Yang et al. (2018)
Sesquiterpene β-Caryophyllene	In vitro-PANC-1, HCT 116, HT29	Not specified	PANC-1: IC50 = 27 μM HCT 116: IC50 = 19 μM HT29: IC50 = 63 μM	Dahham et al. (2015)

### 5.6 Anti-diabetic effect

As a key enzyme in glucose metabolism, α-glucosidase has the dual role of hydrolysis and transglycosidation in the catalytic reaction of sugar. Inhibition of α-glucosidase can effectively improve the efficiency of glucose absorption in the intestine, ameliorate postprandial blood glucose levels, and exert effects in delaying the progression of diabetes. The active component of agarwood, 2-(2-phenylethyl)chromone derivatives, possesses significant inhibitory effects on α-glucosidase (IC50 7.8 ± 0.3–137.7 ± 3.0 μM), which is more potent than acarbose (IC50 743.4 ± 3.3 μM) (Mi et al., 2021). Sesquiterpenes in agarwood also exhibit inhibitory effects on α-glucosidase, with effects superior to acarbose (Mi et al., 2019; Yang et al., 2019). Furthermore, phenethyl chromone compounds from agarwood may improve diabetes by modulating adiponectin secretion (Ahn et al., 2019) (Table 8).

**TABLE 8:** Anti-diabetic effects of Agarwood.

Compound	Experiment	Results	References
2-(2-Phenylethyl)chromone	In vitro-inhibitory effect on α-glucosidase	IC50 = 7.8 ± 0.3–137.7 ± 3.0 μM (acarbose:IC50 = 743.4 ± 3.3 μM)	Mi et al. (2021)
Sesquiterpenoids	In vitro-inhibitory effect on α-glucosidase	IC50 = 253.2 ± 9.7 μM (Acarbose, IC50 = 743.4 ± 3.3 μM)	Mi et al. (2019)
Sesquiterpenoids and 2-(2-phenylethyl)chromones	In vitro-inhibitory effect on α-glucosidase	IC50 = 112.3 ± 4.5–524.5 ± 2.7 µM (acarbose, IC50 = 743.4 ± 3.3 µM)	Yang et al. (2019)

### 5.7 Acetylcholinesterase inhibitory effect

Acetylcholinesterase (AChE) is responsible for the breakdown of acetylcholine at synaptic junctions, terminating the excitatory action of the neurotransmitter on the postsynaptic membrane. The deficiency of acetylcholine is a primary cause of Alzheimer’s disease, and the inhibition of AChE can slow the progression of the condition. The compound 5,6,7,8-Tetrahydro-2-(2-phenylethyl)chromones, isolated from agarwood, has been found to exhibit potent inhibitory effects against AChE, with an inhibition rate of up to 47.9% (with tacrine as a positive control, showing an inhibition rate of 66.7%) (Liao et al., 2017). Epoxy-5,6,7,8-tetrahydro-2-(2-phenylethyl) chromones, isolated from agarwood by Li, also demonstrated significant inhibitory activity, with an AChE inhibition rate of 47.9% ± 0.3% at a concentration of 50 μg/mL (Li et al., 2019). In contrast, 2-(2-phenylethyl) chromone derivatives isolated by Chen, Yang, and others from agarwood exhibited weaker inhibitory activity, with inhibition rates ranging from 12.0% to 15.7% (Yang et al., 2017; Yang et al., 2014) (Table 9).

**TABLE 9:** Acetylcholinesterase inhibitory effects of Agarwood.

Compound	Experiment	Results	References
5, 6, 7, 8-Tetrahydro-2-(2-phenylethyl)chromone	In vitro-iAChE inhibitory activity	50 μg/mL, inhibition ratios = 15.8%, 35.9% and 47.4%, (Tacrine, inhibition ratios = 66.7 ± 0.8)	Liao et al. (2017)
Epoxy-5, 6, 7, 8-tetrahydro-2-(2-phenylethyl)chromones	In vitro-iAChE inhibitory activity	50 μg/mL, IC50 = 441.6 and 155.6 μM (Tacrine IC50 = 0.152 μM)	Li et al. (2019)
Bi-2-(2-phenylethyl)chromone	In vitro-Ellman’s colorimetric method	Inhibition ratios in the range of 12.0%–16.0% (Tacrine, inhibition ratio = 72.8 ± 0.8)	Yang et al. (2017)
2-(2-phenylethyl)chromone	In vitro-Ellman’s colorimetric method	50 μg/mL, inhibition ratios = 20.3% and 25.4% (Tacrine, inhibition ratios = 60.4 ± 0.8)	Yang et al. (2014)

### 5.8 Other pharmacological effects

In addition to the aforementioned pharmacological effects, agarwood possesses various other pharmacological properties, including immune modulation, neuroprotection, and gastrointestinal protection.

The agarwood epoxy compound 2-(2-phenylethyl) chromone derivative (GYF-21) can inhibit both innate and adaptive immune responses by suppressing the STAT1/3 and NF-κB signaling pathways, making it a potential candidate for the treatment of multiple sclerosis (Guo et al., 2017). GYF-21 can also inhibit B cells’ activation, proliferation, and differentiation functions by inhibiting the BAFF signaling pathway, offering potential therapeutic effects for systemic lupus erythematosus (Guo et al., 2019). Furthermore, the sesquiterpene derivative from agarwood (HHX-5) can regulate immune responses by inhibiting macrophage activation, CD4^+^ T cell differentiation, and B cell activation by suppressing the STAT pathway (Zhu et al., 2016b). Also, the hybrid of agarwood 2-(2-phenylethyl)chromone-sesquiterpene can protect gastric mucosal cells by downregulating endoplasmic reticulum stress induced by taurocholic acid (Zhang et al., 2023). The dimer of sesquiterpenes from agarwood wood also exhibits antimalarial effects (IC50 21.67 ± 1.25 nM), with artemisinin serving as a positive control (IC50 559.4 ± 66.55 nM) (Huang X. L. et al., 2023). Agarwood also has therapeutic effects in treating cardiovascular diseases, where 2-(2-phenylethyl) chromone derivatives can mitigate atherosclerosis by reducing endoplasmic reticulum stress-mediated macrophage CD36 expression and inhibiting foam cell formation (Ma et al., 2024).

## 6 Methods of agarwood tree leaves extraction

Agarwood tree leaves have a wide range of pharmacological effects. However, the extraction process of agarwood tree leaves is not the focus of this article, so only a brief introduction to the relevant extraction processes is provided below. Wang extracted the essential oil from agarwood tree leaves using steam distillation, supercritical CO2 extraction, and enzymatic treatment combined with supercritical extraction. The essential oil yields from these three processes were 0.065%, 0.142%, and 0.425%, respectively (Wang et al., 2014). Li used the leaves of “Qi-Nan” agarwood as raw material and ethanol as the solvent, employing an ultrasonic-assisted method to extract flavonoids. The study found that a material-liquid ratio of 1:50, an ethanol concentration of 60%, and an ultrasonic extraction time of 30 min were the ideal conditions for extracting flavonoids. At this time, the content of flavonoids in the extract was 6.68%. The flavonoids had strong antioxidant capacity, with DPPH and ABTS radical scavenging IC50 values of 12.64 μg/mL and 66.58 μg/mL, respectively (Li et al., 2024). Zhang conducted comparative experiments on the anti-inflammatory activity of agarwood tree leaves extracts using different solvents (water, diethyl ether, and normal butanol). It was found that the normal butanol extract had the most potent anti-inflammatory activity. After resin filtration and separation of the normal butanol extract, HPLC detection revealed the highest content of mangiferin, suggesting that mangiferin could serve as a quality control standard for agarwood tree leaf extracts (Zhang, 2014). Using an ultrasonic extraction method, Liu explored the optimal extraction process for mangiferin from agarwood tree leaves. The results indicated that an extraction time of 7 min, microwave ultrasonic power of 408 W, and a material-liquid ratio of 1:44 maximized the extraction rate of mangiferin, which could reach up to 3.229% (Liu M. et al., 2020) (Table 10).

**TABLE 10:** Other pharmacological effects of Agarwood.

Compound	Experiment	Pathways	Results	References
Epoxide 2-(2-Phenethyl)-Chromone	In vivo-male C57BL/6 and BALB/c mice In vitro-BV-2 cells, murine microglia cell line	↓ NF-κB and STAT1/3 Signaling Pathways	↓ TNF-α, IL-1β, IFN-γ, IL-6, MIP-1α, MCP-1 ↓ CD80 and CD86 on CD11c dendritic cells ↓ the phosphorylation of p65, I-κB and STAT1/3,	Guo et al. (2017)
Epoxide 2-(2-Phenethyl)-Chromone	In vivo-BALB/c mice In vitro-Splenocytes	↓ BAFF signaling pathways	↓ Survival, activation, proliferation, and differentiation of B cells stimulated by B-cell activating factor (BAFF)	Guo et al. (2019)
Sesquiterpene	In vivo-BALB/c mice In vitro-RAW264.7	↓ STAT signaling pathways	↓ IL-6, IL-1β, TNF-α, NO ↓ Differentiation of naive CD4, T cells into Th1, Th2, and Th17 cells ↓ Activation, proliferation and differentiation of B cells and CD8 T cells	Zhu et al. (2016b)
2-(2-phenylethyl)chromone-sesquiterpene hybrids	In vitro-GES-1 cell	↓ Perk/eIF2α/CHOP signal pathway	↓ Apoptosis induced by taurocholic acid ↑ GES-1 cell viability ↓ CHOP, P-Perk, P-eIF2α	Zhang et al. (2023)
Sesquiterpene Dimer	In vitro-MCF-7, HEPG2, KYSE30, A549, BGC-823 and the normal cell line LO2	Not specified	↓ Proliferation and migration activity of KYSE30 and BGC-823 cells Plasmodium falciparum 3D7 strain, IC50 = 21.67 ± 1.25 nM (artemisinin, IC50 = 559.4 ± 66.55 nM)	Huang et al. (2023b)
2-(2-Phenylethyl)chromone	In vivo-male C57BL/6J ApoE−/−mice In vitro-THP-1 human monocyte cells	↓ ER stress/P-JNK/PPARγ/CD36 signaling pathway	↓ Therosclerotic lesions in the ApoE−/−mice ↓ oxLDL uptake of macrophages ↓ CD36 gene Expression ↓ CD36 expression by PPARγ ↓ Phosphorylation levels of JNK1/2/3 ↓ oxLDL-induced ER stress	Ma et al. (2024)

## 7 Pharmacological effects of agarwood tree leaves

**FIGURE 2:**
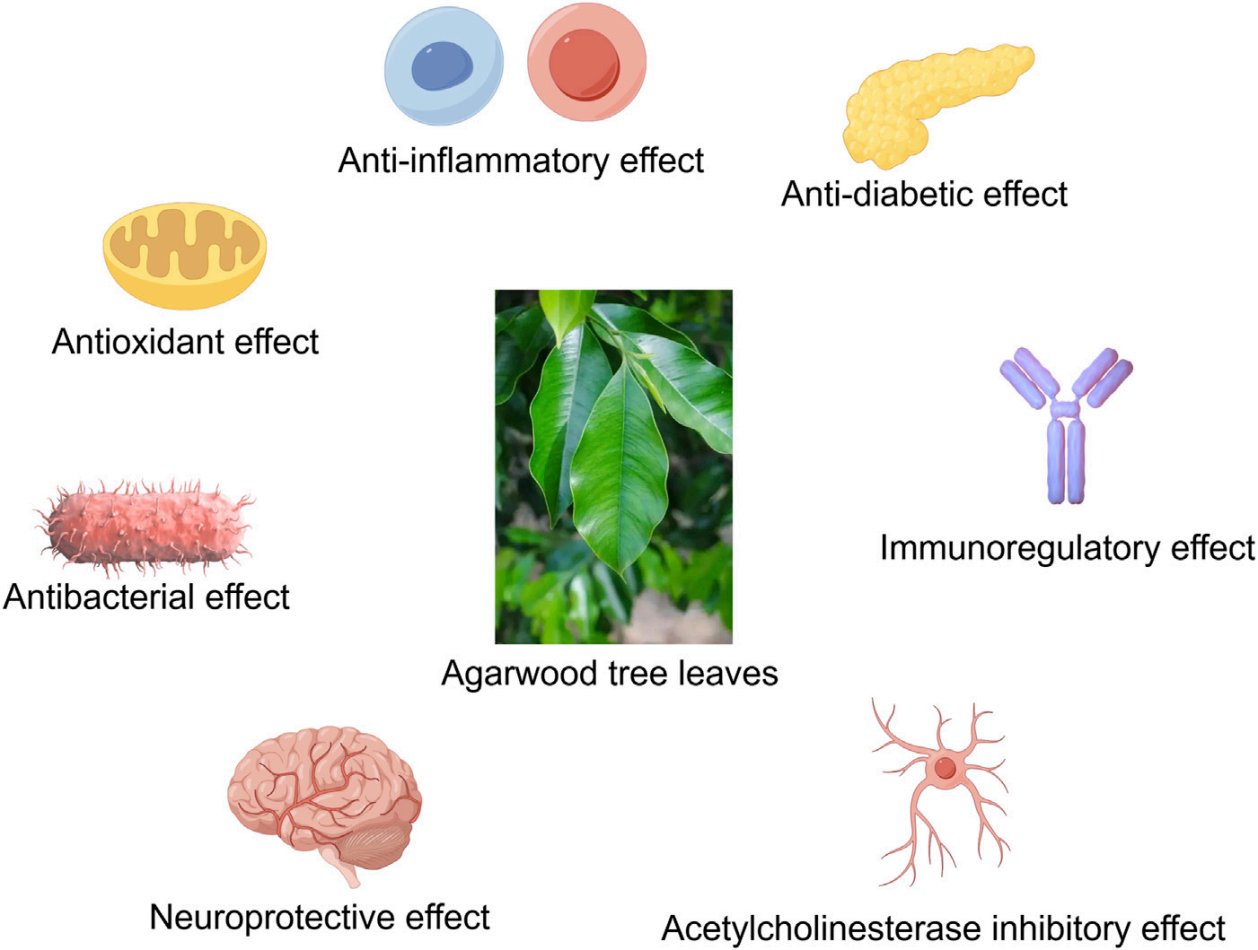
The agarwood tree leaves have anti-inflammatory, antioxidant, antibacterial, neuroprotective, anti-diabetic, immune regulation, acetylcholinesterase inhibition and other effects (By Figdraw).

Agarwood is renowned for its diverse pharmacological activities, and agarwood tree leaves, rich in bioactive compounds such as 2-(2-phenylethyl)chromones, flavonoids, phenolic acids, flavones, and terpenoids, have demonstrated significant anti-inflammatory, antioxidant, immunomodulatory, antibacterial, and antidiabetic effects (Tay et al., 2014; Adam et al., 2017). Studies have shown that agarwood leaf extracts can markedly alleviate ear edema induced by dimethyl benzene, paw edema caused by carrageenan, and leukocyte migration triggered by CMC-Na, exhibiting pronounced anti-inflammatory and analgesic properties (Zhou et al., 2008). Furthermore, agarwood leaf extracts have been found to reduce NO production in macrophages stimulated by LPS, with flavonoid compounds likely being the primary active constituents (Yang et al., 2012; Qi et al., 2009). Research by Pang et al. (2023) has further revealed that agarwood leaf extracts can effectively modulate the immune response mediated by macrophages by regulating the expression levels of TNF-α, IL-6, COX2, and IL-10, as well as by decreasing the expression of iNOS. Regarding antibacterial activity, Hendra’s research has discovered that agarwood tree leaves possess inhibitory effects against *Staphylococcus aureus* and *Escherichia coli*, with mature leaves showing superior antibacterial effects compared to young leaves (Hendra et al., 2016a). The mechanism of action may be related to the disruption of bacterial cell wall synthesis and the inhibition of biofilm formation (Kamonwannasit et al., 2013). Agarwood leaf extracts also exhibit α-glucosidase inhibitory activity, thereby reducing blood glucose levels, akin to the effects of agarwood itself (Yang et al., 2024; Feng et al., 2011). Extracts from Aquilaria crassna leaves can also counteract the neurotoxicity and aging effects of hyperglycemia, with stigmasterol and β-sitosterol being potential active components (Pattarachotanant et al., 2022). Besides, the active component Clionasterol in Aquilaria crassna leaves has been shown to protect neuronal cells and *Caenorhabditis elegans* from toxic damage (Pattarachotanant et al., 2023). Regarding the safety of agarwood leaf extracts, studies have indicated that no significant toxic reactions or deaths were observed in mice administered a dose of 2000 mg/kg for 14 consecutive days, and no abnormalities were found in pathological examinations (Kamonwannasit et al., 2013). Nevertheless, further research is warranted to confirm the safety profile of agarwood leaf extracts (Table 11; Figures 2).

**TABLE 11:** Pharmacological effects of agarwood tree leaves.

Pharmacological effects	Compound	Experiment	Pathways	Results	References
Anti-inflammatory	Aquilaria sinensis (Lour.) Gilg. Leaves extract	In vivo-ICR Mice	Not specified	↓ Acetic acid-induced writhing, and the response to the thermal stimulus ↓ Xylene-induced ear swelling, CMC-Na-induced leukocyte migration, carrageenan-induced paw edema ↓ NO production (IC50 = 80.4 mg/mL)	Zhou et al. (2008)
	Aquisiflavoside from the leaves of Aquilaria sinensis	In vitro-RAW264.7	Not specified	↓ NO production (IC50 = 34.95 μM)	Yang et al. (2012)
	Aquilaria malaccensis Leaf Extract	In vitro-RAW264.7	Not specified	↓ NO production	Eissa et al. (2022)
Antioxidant	Polyphenols from agarwood (Aquilaria crassna)	In vitro-2, 2-diphenyl-1-picrylhydrazyl (DPPH) radical scavenging test	Not specified	DPPH radical scavenging capacity = 93.7%	Tay et al. (2014)
	Agarwood (Aquilaria malaccensis Lamk.) leaves	In vitro-DPPH radical scavenging test	Not specified	IC_50_ = 19.62 ± 1.49 μg/mL	Hendra et al. (2016b)
	Aquilaria crassna leaf extract	In vitro-ABTS, FRAP and DPPH scavenging methods	Not specified	DPPH: IC50 = 7.25 ± 2.05 μg/mL (ascorbic acid: IC50 = 1.33 ± 0.08 μg/mL) ABTS: IC50 = 218.93 ± 29.77 μg/mL (BHT: IC50 = 83.09 ± 0.45 μg/mL) FRAP = 1.18 ± 0.07 μmol of Fe^2+^/mg dried extract.	Kamonwannasit et al. (2013)
	Aquilaria sinensis (Lour.) Gilg leaves	In vitro-ABTS and FRAP scavenging methods	Not specified	ABTS: IC50 = 0.366 (Trolox E: IC50 = 0.137 ± 0.06) FRAP:IC50 = 0.833 mg/mL (FeSO4·7H2O: IC50 = 0.398 ± 0.001 mg/mL)	Yang et al. (2024)
Immunomodulatory effects	Agarwood Leaf Extract	In vitro-RAW264.7	Not specified	↑ TNF-α, IL-6, IL-10, COX-2 ↓ iNOS	Pang et al. (2023)
Antibacterial	agarwood (Aquilaria malaccensis Lamk.) leaves	In vitro- disc diffusion assay	Not specified	*S. aureus*, diameter of inhibition zone = 10.83 mm *E. coli*, diameter of inhibition zone = 9.92 mm	Hendra et al. (2016a)
	Aquilaria crassna leaf extract	In vitro- disc diffusion assay	Not specified	S. Epidermidis, Diameters of inhibition zone = 12.0–18.0 mm (Vancomycin 30 μg, inhibition zone = 21.0 mm) MIC = 6 mg/mL MBC = 12 mg/mL (Vancomycin, MIC = 1.5 μg/mL, MBC = 3.0 μg/mL)	Kamonwannasit et al. (2013)
Anti-diabetic	Aquilaria sinensis (Lour.) Gilg leaves	In vitro-α-glucosidase inhibitory assay	Not specified	EC50 = 0.844 mg/mL (acarbose, EC50 = 0.183 mg/mL)	Yang et al. (2024)
	Aquilaria sinensis leaves	In vitro-α-glucosidase inhibitory assay	Not specified	IC50 = 126.5–312.3 μg/mL (acarbose, IC50 = 372.0 μg/mL)	Feng et al. (2011)
	Aquilaria leaves	In vitro-α-glucosidase inhibitory assay	Not specified	IC50 = 0.88–1.23 μg/mL (acarbose, IC50 = 213.14 μg/mL)	Ito and Ito (2022)
Acetylcholinesterase Inhibitory	Aquilaria subintegra	In vitro-AChE inhibitory activity	Not specified	Inhibition ratios = 80%	Bahrani et al. (2014)
Neuroprotective effect	Aquilaria crassna Leaf Extract	In vivo-nematode *Caenorhabditis elegans* In vitro-human neuroblastoma SH-SY5Y cells	↓ D1/SIRT1 signaling pathway	↓ Cyclin D1 and SIRT1 ↓ ROS ↑ The mean lifespan of high glucose-fed worms ↑ DAF-16, SOD-3, AQP-1	Pattarachotanant et al. (2022)
	Aquilaria crassna Leaf Extract	In vivo-nematode *Caenorhabditis elegans* In vitro-human neuroblastoma SH-SY5Y cells	↓ AHR/CYP1A1/CCND1 signaling pathway	↑ Percentage of cells in the G0/G1 phase ↓ AHR, CYP1A1, cyclin D1 ↑ Mean lifespan of high Benzo [a]pyrene-fed worms ↓ Toxic effect of Benzo [a]pyrene ↓ CYP-35A1, CYP-35A2, CYP-35A3, CYP-35A4, CYP-35A5, CYP-35B, CYP-35B3, CYP-35C1 ↓ Hxk-1, hxk-2, hxk-3	Pattarachotanant et al. (2023)
	Aquilaria crassna leaves	In vivo-male Sprague Dawley rats	Not specified	↑ Relaxed mesenteric artery	Wisutthathum et al. (2019)

## 8 Research on the correlation between agarwood and periodontitis

**FIGURE 3:**
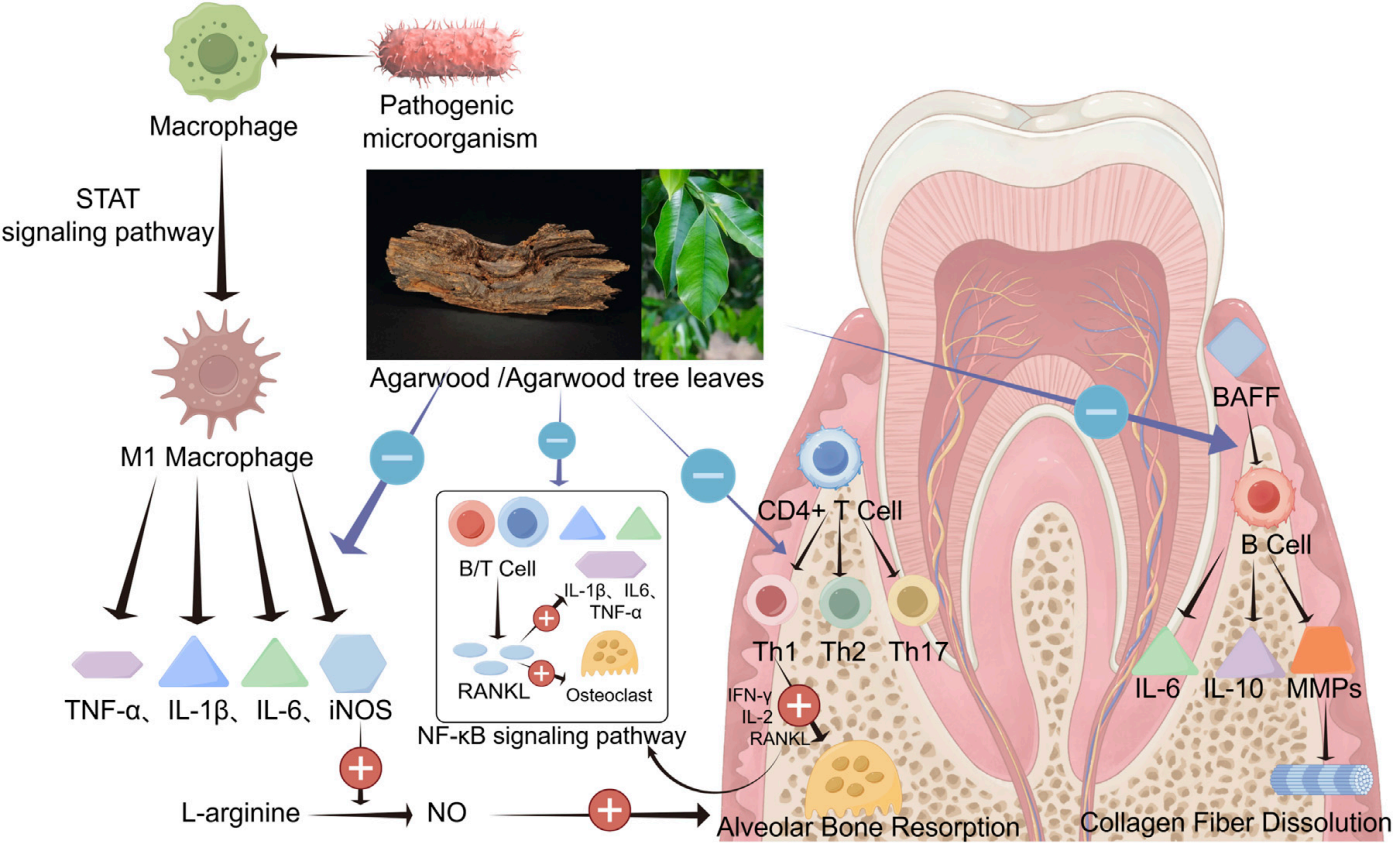
Agarwood and agarwood tree leaves own a strong role and broad prospects in the treatment of periodontitis. The various active substances in agarwood can inhibit or downregulate inflammation levels in periodontal tissue, reduce immune cell differentiation and activation, prevent periodontal tissue destruction, and inhibit the progression of periodontal inflammation by suppressing signaling pathways such as NF-κB and STAT (By Figdraw).

Although dental plaque is the initiating factor of periodontitis, the host’s immune response is crucial in destroying periodontal tissues. Macrophages, CD4^+^ T cells, and B cells mediate both Innate and adaptive immune responses, and signaling pathways such as receptor activator of NF-κB ligand (RANKL) and STAT 1, along with inflammatory factors like TNF-α, IL-1β, IL-6, PGE2, play significant roles in the progression of periodontal inflammation. Chronic inflammation can lead to irreversible tissue damage. As mentioned above, agarwood possesses a wide range of pharmacological effects, including potent anti-inflammatory, antioxidant, and immunomodulatory properties. Inflammation, oxidative stress, and immune imbalance are the primary pathological responses in periodontitis, suggesting that agarwood holds great potential in treating periodontal disease. Concurrently, our preliminary experimental research has found that agarwood extracts can effectively reduce attachment loss in rats with periodontitis and improve the biological parameters of alveolar bone Figures 3.

### 8.1 Immune cells

Macrophages play a crucial role in maintaining homeostasis and defense in periodontal tissues. However, their excessive aggregation and activation can damage periodontal tissue. Under the stimulation of pathogenic microorganisms and inflammatory cells, macrophages can polarize into M1 type macrophages through the STAT 1 signaling pathway (Han et al., 2020; Wang W. et al., 2021), and secrete inflammatory factors such as PGE2, TNF-α, IL-1β, IL-6, and iNOS. PGE2 is the most potent cytokine in promoting periodontal bone destruction, which can mediate destructive processes of alveolar bone and cementum by downregulating the mineralization ability of osteoblasts and promoting osteoclast formation (Oka et al., 2007). TNF-α, IL-1β, and IL-6 primarily stimulate the chemotaxis and migration of inflammatory cells to periodontal tissues, enhance the expression of neutrophil adhesion factors on the vascular surface, and augment the synthesis of other pro-inflammatory cytokines. They also exert destructive effects on bone and connective tissues by promoting the expression of MMPs and RANKL (Cekici et al., 2014). iNOS can catalyze NO production from L-arginine until the substrate is depleted (Fretwurst et al., 2020). Low NO concentrations within the periodontal tissue are beneficial for exerting anti-inflammatory effects. In contrast, high NO concentrations can exacerbate inflammatory responses caused by bacteria and endotoxins, accelerating the destruction of alveolar bone (Sun S. et al., 2020). Zhu investigated the anti-inflammatory activity of agarwood 2-(2-phenylethyl)chromone derivative (GYF-17) against LPS-induced RAW264.7 cells and its potential mechanisms, and the results showed that GYF-17 could reduce the expression of iNOS induced by LPS and downregulate the release of NO. In addition, GYF-17 significantly inhibited the expression and secretion of vital pro-inflammatory factors such as IL-1β, IL-6, PGE2, and TNF-α (Zhu et al., 2016a). Wang’s research indicates that agarwood extract can effectively reduce the expression levels of inflammatory cytokines such as TNF-α and IL-6 (Wang et al., 2023).

In states of inflammation, CD4^+^ T cells in the periodontal tissue can proliferate and differentiate into various subtypes of Th cells, such as Th1, Th2, and Th17. Th1 cells, characterized by their role in cell-mediated immunity, can lead to alveolar bone resorption and promote the progression of periodontal inflammation by releasing IFN-γ, IL-2, and RANKL. Th17 cells produce vital cytokines such as IL-17, which plays a positive role in bone remodeling and homeostasis (Yu et al., 2007). However, excessive IL-17 production can exacerbate periodontal tissue destruction by upregulating the expression of chemokine ligands (CXCL1, CXCL2, and CXCL5), IL-1β, MMPs, granulocyte colony-stimulating factors (G-CSF) and granulocyte-macrophage colony-stimulating factors (GM-CSF) (Veldhoen, 2017). The upregulation of G-CSF and GM-CSF increases tissue permeability, mediating chemotaxis and activation in periodontal tissues, thereby exacerbating damage through phagocytosis and cellular immunity.

In periodontitis patients, the proportion of B cells in the lesion tissue correlates positively with the severity of periodontitis (Reinhardt et al., 1988). Under the stimulation of pathogenic microorganisms, B cells perform antigen presentation to trigger immune responses in CD4^+^ T cells and differentiate themselves into plasma cells that secrete immunoglobulins to neutralize antigens. B cells can also produce inflammatory cytokines such as TGF-β, TNF-α, IL-10, and IL-6 to modulate inflammatory responses and generate matrix metalloproteinases (MMPs), which are crucial in physiological and pathological processes. Collagenases like MMP-1 and MMP-8 can dissolve types I, II, and III collagen fibers, leading to collagen dissolution and periodontal connective tissue destruction (Zou et al., 2022; Nędzi-Góra et al., 2021; Luchian et al., 2022). B cell activating factor (BAFF) plays a critical regulatory role, enhancing B cell survival, proliferation, and antigen presentation by binding to receptors, including BAFFR, and promoting plasma cell differentiation. As mentioned earlier, agarwood possesses significant immunomodulatory functions, and its active components could potentially serve as a source of immunosuppressive agents. The sesquiterpene derivative from agarwood (HHX-5) can effectively inhibit the activation, proliferation, and differentiation of CD4^+^ and CD8^+^ T cells, thereby suppressing cell-mediated immune responses (Zhu et al., 2016b). Furthermore, the 2-(2-phenylethyl) chromone derivative from agarwood (GYF-21) can inhibit B cells’ activation, proliferation, and differentiation by blocking the signaling pathway activated by BAFF (Guo et al., 2019).

### 8.2 Signaling pathways

#### 8.2.1 NF-κB signaling pathway

The NF-κB signaling pathway has long been recognized as an essential and common pro-inflammatory signaling pathway. Within this pathway, RANKL is a crucial activator. Under its stimulation, NF-κB-induced mitogen-activated protein kinase is activated, phosphorylating protein kinase NIK and its downstream IKKα, which leads to the phosphorylation and degradation of NF-κB p100, forming dimers such as RelB/p52. These components enter the nucleus to regulate the transcription of target genes, causing cells to produce corresponding inflammatory and stress responses (Sun, 2011). RANKL is also a major regulatory factor for the differentiation and function of osteoclasts, with its primary function being the activation of osteoclasts and the upregulation of the expression of pro-inflammatory cytokines TNF-α, IL-6, and IL-1β. Periodontal tissues, including the gingiva, alveolar bone, cementum, and periodontal ligament, all of which can express RANKL (Usui et al., 2021). Under inflammatory conditions, cells of the periodontal tissues, B lymphocytes, and activated T cells can release large amounts of RANKL and pro-inflammatory factors. The aforementioned inflammatory pathways and mechanisms promote the development of inflammation within the periodontal tissues, upregulate the production of osteoclasts, and subsequently cause the resorption of alveolar bone (Fischer and Haffner-Luntzer, 2022). Inhibition of RANKL release would be beneficial for the protection of periodontal tissues. Studies have indicated that pretreatment with ethanol extracts of agarwood can suppress the activation of RANKL and reduce the expression of TNF-α, IL-6, and IL-1β (Wang C. et al., 2021). GYF-21 has been shown to exert significant anti-inflammatory effects by inhibiting the NF-κB signaling pathway (Guo et al., 2019), which suggests that agarwood may inhibit the progression of periodontal inflammation by suppressing the RANKL pathway.

#### 8.2.2 STAT signaling pathway

In inflammatory cytokine and signaling molecule production, the STAT is a primary regulatory component. STAT 1, as a crucial regulatory factor in inflammatory responses, is involved in processes that inhibit cell proliferation and differentiation. Under inflammatory stimuli, the expression level of STAT 1 in periodontal ligament cells significantly increases compared to the control group. High levels of STAT 1 have a marked inhibitory effect on the osteogenic differentiation capability of periodontal ligament cells, disrupting the osteogenic-osteoclastic balance and leading to the destruction and resorption of supportive tissues such as alveolar bone (Lin et al., 2023; Kim et al., 2003). Additionally, the osteogenic gene Runt-related transcription factor 2 (Runx2) can promote osteoblast differentiation. Meanwhile, STAT 1, acting as a negative regulator of Runx2, can inhibit Runx2 from entering the nucleus, thereby preventing the expression of osteogenic differentiation genes (Kim et al., 2003). Beyond its negative impact on osteogenesis, STAT1 also promotes the polarization of macrophages to the M1 phenotype. LPS-induced activation of Toll-like receptor 4 (TLR4) via the JAK2/STAT1 pathway is critical for M1 polarization. LPS induces interferon-β, which facilitates the formation of STAT1-STAT2 heterodimers, leading to the assembly of the IFN-stimulated gene factor 3 complex and regulation of M1-associated gene expression (Li et al., 2018). The presence of numerous M1 macrophages in periodontal tissues can result in inflammatory responses characterized by cytotoxicity and tissue damage.

Apart from STAT1, STAT3 also plays a significant role in the progression of periodontitis. STAT3, an integral component of the JAK/STAT signaling pathway, is highly phosphorylated in inflammatory tissues and crucial for regulating the expression of cytokines, chemokines, and other mediators that induce and sustain an inflammatory environment (Garama et al., 2015). Research by Song indicates that the knockdown of STAT-3 suppresses IL-1β-induced expression of MMP-1 and MMP-3 (Song et al., 2021). STAT3 also acts as a sensor for various metabolic stressors, including ROS. Phosphorylated STAT3 translocates from the cytoplasm to the nucleus, where it functions as a transcription factor, inducing the expression of downstream oxidative stress and inflammatory factors, thereby causing further tissue damage (Lu et al., 2019). Studies suggest that inhibiting this signaling can control the expression of inflammatory factors and ROS (Li et al., 2020).

In summary, STAT proteins in periodontal tissues can exert destructive effects by inhibiting periodontal osteogenesis, promoting macrophage polarization, pro-inflammatory actions, and oxidative stress. Research has indicated that HHX-5 can significantly inhibit the activation of the STAT 1 signaling pathway in macrophages and CD4^+^ T cells under inflammatory stimuli (Zhu et al., 2016b), and GYF-17 can suppress the progression of inflammation by inhibiting the STAT 1/3 and ERK 1/2 pathways, suggesting that agarwood may act through the STAT 1 pathway to inhibit periodontitis (Zhu et al., 2016a).

## 9 Research progress of agarwood essential oils nanoemulsions

Plant essential oils are a common form of plant extracts, primarily composed of hydrocarbons such as monoterpenes and sesquiterpenes, as well as aromatic compounds like alcohols, aldehydes, and ketones (Tongnuanchan and Benjakul, 2014). Numerous studies have demonstrated the broad pharmacological effects of plant essential oils, including antioxidant, anti-inflammatory, and analgesic properties (Silva et al., 2015). However, after ingestion, plant essential oils rapidly undergo glucuronidation and are metabolized and eliminated by the urinary or respiratory systems. Their short half-life limits the promotion of their clinical applications (Kohlert et al., 2000).

As an advanced drug delivery system, nanoemulsion has been proven to enhance solubility or bioavailability effectively. The tiny droplets in nanoemulsion, typically with diameters ranging from 10 to 100 nm, can increase the total surface area, thereby improving the absorption efficiency of drugs (Singh et al., 2017). By encapsulating drugs in oily or aqueous materials according to the properties and intended use of the drug, their stability can be enhanced, preventing premature or rapid degradation in the body, thus achieving the goal of increasing drug bioavailability (Deshmukh et al., 2021). Research by Liang et al. has shown that cinnamon essential oil nanoemulsions remain undigested in the mouth, with the digestion process mainly occurring in the stomach and small intestine, demonstrating the potential of nanoemulsion to effectively enhance drug bioavailability (Liang et al., 2022).

As previously mentioned, agarwood possesses a broad range of pharmacological effects, including potent anti-inflammatory, antioxidant, antitumor, antimicrobial, and hypoglycemic properties. However, the bioactive components of agarwood are primarily present in the form of essential oils, which results in poor solubility and low bioavailability, limiting its clinical applications. Advanced drug delivery systems can effectively address this issue, and among them, nanoemulsions have emerged as a preferred solution due to their simple preparation process, strong stability, and good biocompatibility (Jamir et al., 2024; Shetta et al., 2024).

Currently, no research compares Agarwood nanoemulsion’s bioavailability and stability with traditional Agarwood essential oils. Research on agarwood nanoemulsions has primarily focused on respiratory system diseases, with no reports found related to the oral field. Malik’s research revealed that agarwood nanoemulsions effectively inhibited the expression of inflammatory factors TNF-α, IL-1β, IL-6, and iNOS while also reducing the secretion of ROS and NO levels, demonstrating anti-inflammatory solid and antioxidant effects (Malik et al., 2023). Alamil found that agarwood essential oil nanoemulsions could downregulate the expression of IL-6 and IL-8 in bronchial epithelial cells stimulated by inflammatory mediators (Alamil et al., 2023). Rubis’s research indicated that agarwood essential oil nanoemulsions possess anti-inflammatory and antioxidant properties, capable of inducing the expression of antioxidant genes such as GCLC and GSTP1 and downregulating the expression of pro-inflammatory factors like IL-8, IL-1α, IL-1β, as well as upregulating the expression of anti-inflammatory mediators IFN-γ, IL-10, IL-18BP, and GH, suggesting their significant potential in treating chronic obstructive pulmonary disease (COPD) (De Rubis et al., 2023). In summary, it is suggested that Agarwood nanoemulsion also possesses significant anti-inflammatory and antioxidant effects in the oral field. Moreover, because nanoemulsion technology can mprove drug stability and bioavailability, Agarwood nanoemulsion may be more effective than traditional Agarwood essential oils, indicating a broad application prospect.

## 10 Conclusion and prospects

Agarwood has long captivated people due to its rich bioactive components, extensive pharmacological effects, and unique aromatic scent. However, the high demand for agarwood, its long production time, low yield, and inconsistent quality have led to a persistent shortage and high prices. Currently, induction methods for agarwood formation are categorized into natural formation and traditional induction, biological induction, and chemical induction. Among these, chemical induction stands out as the most ideal and reliable alternative to natural agarwood formation because it can stably induce the formation of high-quality agarwood and effectively addresses the issue of poor repeatability in the quality of artificially induced agarwood. However, due to the limited research conducted thus far, extensive experimentation is still required to verify the safety and feasibility of chemical induction before it can be widely adopted. Regarding the distillation of agarwood essential oil, supercritical fluid extraction (SFE) and subcritical water extraction (SCWE) are currently preferred methods. These techniques offer several advantages, including high extraction efficiency, stable oil quality, and a rich content of active components. As research in this field continues to advance, it is expected that more efficient and environmentally friendly methods for agarwood production and essential oil extraction will be developed, further expanding the applications and availability of this precious resource.

Agarwood is rich in sesquiterpenes, 2-(2-phenylethyl) chromone derivatives, aromatic compounds, and fatty acids, which endow it with a broad spectrum of pharmacological effects, such as anti-inflammatory, antioxidant, immunomodulatory, hypoglycemic, and antitumor properties. Since inflammatory responses, oxidative stress, and immune dysregulation are the primary pathological mechanisms of periodontitis, agarwood essential oil suggests significant potential in treating periodontal disease. However, despite being abundant in bioactive components of agarwood, essential oils suffer from drawbacks such as low solubility and poor bioavailability, which limit their clinical applications. Therefore, improving the active form is crucial. Nano-encapsulated drug delivery systems are an effective measure to address these issues. Existing researchs have demonstrated that agarwood nanoemulsion possesses potent anti-inflammatory and antioxidant effects. Perhaps using agarwood nanoemulsions to treat periodontitis is a feasible strategy. However, more *in vivo* and *in vitro* experiments are needed to substantiate the feasibility of the experiments, drug dosage, and potential toxicity issues.

The research focus on agarwood is predominantly centered on the pharmacological activities of its resin components and their applications in clinical medicine. In comparison, research on the leaves of agarwood trees has been relatively scarce. However, existing research findings indicate that agarwood tree leaves are rich in pharmacologically active constituents. They hold potential therapeutic value and present unique advantages in resource utilization and medicinal research due to their lower economic cost and relatively more straightforward acquisition. Therefore, agarwood tree leaves have the potential to emerge as a new hotspot in future research. The findings in this area are expected to bring innovative breakthroughs to the pharmaceutical and health industries.

## Nomenclature

### Resource identification initiative

To take part in the Resource Identification Initiative, please use the corresponding catalog number and RRID in your current manuscript. For more information about the project and for steps on how to search for an RRID, please click here.

### Life science identifiers

Life Science Identifiers (LSIDs) for ZOOBANK registered names or nomenclatural acts should be listed in the manuscript before the keywords with the following format:

urn:lsid:<Authority>:<Namespace>:<ObjectID>[:<Version>].
